# Variation by Race in Antibiotics Prescribed for Hospitalized Patients With Skin and Soft Tissue Infections

**DOI:** 10.1001/jamanetworkopen.2021.40798

**Published:** 2021-12-23

**Authors:** Alysse G. Wurcel, Utibe R. Essien, Christina Ortiz, Xiaoqing Fu, Christian Mancini, Yuqing Zhang, Kimberly G. Blumenthal

**Affiliations:** 1 Division of Geographic Medicine and Infectious Diseases, Department of Medicine, Tufts Medical Center, Boston, Massachusetts; 2 Tufts University School of Medicine, Boston, Massachusetts; 3 Division of General Internal Medicine, University of Pittsburgh School of Medicine, Pittsburgh, Pennsylvania; 4 Division of Rheumatology, Allergy and Immunology, Massachusetts General Hospital, Boston; 5 Harvard Medical School, Boston, Massachusetts

## Abstract

This cohort study examines antibiotics prescribed and variations by race among hospitalized patients with skin and soft tissue infections (SSTIs).

## Introduction

Antibiotic prescriptions for skin and soft tissue infection (SSTI) are increasing.^1^ SSTI treatment guidelines consider a β-lactam, such as cefazolin, as first-line therapy to target *Staphylococcus aureus* and *Streptococcus* species; alternative SSTI treatments, such as clindamycin, are inferior.^2^ Racial differences in treatment exist for several medical conditions. However, little is known about disparities in SSTI treatment.^3^ We hypothesized that racial differences in the management of SSTI exist.

## Methods

This cohort study conducted a subanalysis of multisite, cross-sectional data collected through a national survey of Acute Care Hospital Groups within Vizient Inc (October 16, 2018, to January 13, 2019) (eMethods in the Supplement), considering adult inpatients treated for SSTI. The indicator of interest was race determined from electronic health record and categorized as Asian, Black, American Indian or Alaska Native, White, or other (which also included those who declined). The outcome was antibiotic use. Mass General Brigham and Tufts institutional review boards reviewed the study and determined it exempt from review and the requirement for informed consent because it was not human participant research. This study followed the Strengthening the Reporting of Observational Studies in Epidemiology (STROBE) reporting guideline. Because the number of individuals identified as Asian (n = 13), American Indian or Alaska Native (n = 5), and other races (n = 146) was small, we only compared the antibiotic use between Black (the referent group) and White individuals. To account for clustering by hospital, we used generalized estimating equations to obtain unadjusted and adjusted odds ratios (aOR) with 95% CI. All *P* values were 2-sided, and *P* ≤ .05 was considered statistically significant. Statistical analysis was performed with SAS software version 9.4 (SAS Institute) in September 2021.

## Results

Of 1242 adult inpatients treated for SSTI from 91 US hospitals, 494 (45%) were female, 224 (18%) were Black, and 854 (69%) were White; the mean (SD) age was 58 (17) years (Table 1). History of penicillin allergy (23% [n = 48] with hives, 19% [n = 38] with rash, 18% [n = 36] with unknown) was more frequent in Black inpatients (23% [n = 52]) vs White inpatients (18% [n = 153]). Piperacillin-tazobactam and vancomycin were most prescribed and did not differ by race. Cefazolin was more commonly used in White inpatients than in Black inpatients (13% [n = 114] vs 5% [n = 11]); clindamycin was more frequently used in Black inpatients than in White inpatients (12% [n = 27] vs 7% [n = 62]) (Table 2). Adjusting for multiple factors, White inpatients were at an increased risk of cefazolin use (aOR, 2.82 [95% CI, 1.41-5.63]) and decreased risk of clindamycin use (aOR, 0.54 [95% CI, 0.30-0.96]) compared with Black inpatients.

**Table 1.** Characteristics of White and Black Adult Inpatients With SSTI at Acute Care Hospitals

Characteristic	Inpatients, No. (%)	
	White (n = 854)	Black (n = 224)
Age, mean (SD), y	60 (17)	56 (17)
Sex		
Female	390 (46)	104 (46)
Male	464 (54)	120 (54)
Inpatient location		
General medical floor	406 (48)	127 (57)
General surgery floor	179 (21)	47 (21)
Adult intensive care unit	57 (7)	9 (4)
Cardiology/telemetry	48 (6)	16 (7)
Oncology	31 (4)	4 (2)
Other	133 (16)	21 (9)
Kidney disease	156 (18)	54 (24)
Diabetes	311 (36)	100 (45)
MRSA colonization/infection	141 (17)	35 (16)
VRE colonization/infection	29 (3)	5 (2)
Allergy to penicillins	153 (18)	52 (23)
Allergy to cephalosporins	62 (7)	7 (3)
Hospital day, median (IQR)	5 (2-8)	5 (2-9)

Abbreviations: MRSA, methicillin-resistant *Staphylococcus aureus*; SSTI, skin and soft tissue infection; VRE, vancomycin-resistant *Enterococcus*.

**Table 2.** Antibiotic Use Among Patients With Cellulitis/Skin or Soft Tissue Infection at Acute Care Hospitals by Race^a^

	White (n = 854)	Black (n = 224)	*P* value, univariable	Unadjusted, OR (95% CI)^b^	Adjusted OR (95% CI)^b^	*P* value^c^
β-lactams						
Piperacillin-tazobactam	132 (15)	32 (14)	.66	1.10 (0.67-1.79)	1.10 (0.65-1.87)	.72
Cefazolin^d^	114 (13)	11 (5)	<.001	2.98 (1.49-5.97)	2.82 (1.41-5.63)	.003
Cefepime	104 (12)	29 (13)	.76	0.93 (0.60-1.44)	0.98 (0.61-1.58)	.94
Penicillins^e^	89 (10)	21 (9)	.65	1.12 (0.68-1.87)	0.98 (0.58-1.66)	.93
Ceftriaxone	76 (9)	20 (9)	.99	1.00 (0.59-1.70)	0.95 (0.56-1.61)	.84
Cephalexin	45 (5)	9 (4)	.44	1.33 (0.73-2.43)	1.29 (0.68-2.44)	.44
Carbapenems^f^	45 (5)	17 (8)	.18	0.68 (0.37-1.24)	0.73 (0.40-1.33)	.30
β-lactam alternatives						
Vancomycin	305 (36)	66 (29)	.080	1.33 (0.97-1.82)	1.26 (0.90-1.77)	.18
Clindamycin^g^	62 (7)	27 (12)	.020	0.57 (0.34-0.97)	0.54 (0.30-0.96)	.04
Tetracyclines^h^	55 (6)	12 (5)	.55	1.22 (0.57-2.61)	1.20 (0.55-2.59)	.65
Fluoroquinolones^i^	49 (6)	19 (8)	.13	0.66 (0.40-1.08)	0.74 (0.44-1.24)	.25

Abbreviation: OR, odds ratio.

^a^ Antibiotics used in less than 5% of the population are not shown. Patients could have been treated with more than 1 antibiotic.

^b^ OR (95% CI) compares antibiotic use in White inpatients with Black inpatients.

^c^ Adjusted for hospital day, penicillin allergy history, intensive care unit location, methicillin-resistant *Staphylococcus aureus* colonization/infection, kidney disease, and diabetes.

^d^ Cefazolin monotherapy was received by 91 White inpatients (11%) compared with 7 Black inpatients (3%) (unadjusted OR, 8.67 [95% CI, 2.70-27.8], *P* < .001).

^e^ Other than piperacillin-tazobactam.

^f^ Includes meropenem (n = 39), ertapenem (n = 19), and imipenem-cilastin (n = 4).

^g^ Clindamycin monotherapy was received by 35 White inpatients (4%) and 13 Black inpatients (6%) (unadjusted OR, 1.79 [95% CI, 0.63-5.10], *P* = .27).

^h^ Includes doxycycline (n = 61) and minocycline (n = 6).

^i^ Includes ciprofloxacin (n = 36), levofloxacin (n = 29), and moxifloxacin (n = 3).

## Discussion

This study found that race was associated with differential management of SSTI. Black inpatients were less likely to receive cefazolin and more likely to receive clindamycin compared with White inpatients. Cefazolin is one of the first-line SSTI treatments. Clindamycin is not recommended given frequent dosing and high potential for adverse effects including *Clostridioides difficile* infection (CDI). Although prior studies of antibiotic choice for infections have revealed practice inconsistent with guidelines,^4,5^ racial differences have not been investigated.

Black race was associated with increased clindamycin use, even after controlling for methicillin-resistant *Staphylococcus aureus* (MRSA) colonization, infection and penicillin allergy. Although penicillin allergy is described as more prevalent in White patients,^6^ we observed an increased prevalence of penicillin allergy in Black inpatients compared with White inpatients treated for SSTI. Given that historical penicillin allergies are associated with increased clindamycin use and risk of CDI, but are often disproved with formal testing,^6^ racial disparities in penicillin allergy documentation and assessment requires additional study.

Although our data came from a large sample of hospitals, these data may not be nationally representative. Race was extracted from the medical record and may have been incorrectly assigned. Other race was not specified, and Hispanic ethnicity was not captured. Data on structural vulnerability such as income, employment, and education level, were not collected, limiting our evaluation of the impact of structural racism. Cross-sectional data did not permit determination of cumulative antibiotic utilization metrics.

We detected a potential racial disparity in antibiotic choice in this study. Future work should evaluate the determinants of this observed difference and devise interventions to achieve pharmacoequity.

## Notes

Supplement.
eMethods
