# Supplementary material for: Variation by Race in Antibiotics Prescribed for Hospitalized Patients With Skin and Soft Tissue Infections
Source: JAMA Netw Open. 2021 Dec 23;4(12):e2140798. doi: 10.1001/jamanetworkopen.2021.40798 (PMC8703249; doi:10.1001/jamanetworkopen.2021.40798)
Supplement: Supplement. — eMethods [file jamanetwopen-e2140798-s001.pdf]

Supplemental Online Content

Wurcel AG, Essien UR, Ortiz C, et al. Variation by race in antibiotics prescribed for hospitalized patients with skin and soft tissue infections. *JAMA Netw Open.* 2021;4(12):e2140798. doi:10.1001/jamanetworkopen.2021.40798

eMethods. Supplemental Methods

This supplemental material has been provided by the authors to give readers additional information about their work.

© 2021 Wurcel AG et al. *JAMA Network Open.*

eMethods. Supplemental Methods

Study Design
We performed a national cross-sectional study of hospitalized patients using the Acute Care Hospital Groups within Vizient. Vizient, Inc is the largest member-driven, health care performance improvement company in the US; over 3,200 acute care hospitals and more than 95% of all US academic medical centers are Vizient members. Vizient, Inc has smaller Acute Care Hospital Groups: 1) The Vizient member pharmacy network contains 1,000 Vizient member hospitals, and 2) the University Health System Consortium pharmacy network contains 120 US academic medical centers and their affiliates. The Acute Care Hospital Groups within Vizient maintains an email listserv of 147 contacts at Vizient member hospitals that was used to identify hospital participants for this study. There were 91 US hospitals that were acute care hospitals that submitted data on a patients with a skin or soft tissue infection. Hospitals had representation from all US census regions, 83 (91%) were in an urban setting and 8 (9%) in a rural setting. There were 55 (60%) community hospitals and 36 (40%) academic medical centers.

Data Collection
Vizient member hospitals who received study information were asked to complete an intake questionnaire; the survey was initially designed by physicians and pharmacists with survey research expertise and later revised based on cumulative feedback from Vizient University Health System Consortium Pharmacy Network's Antimicrobial Stewardship Committee. The questionnaire included fields for hospital details (e.g., type of hospital, antibiotic stewardship team presence) and penicillin allergy assessment resources (e.g., access to inpatient allergy consultations, access to penicillin skin testing). Intake questionnaires were completed from September 18, 2018 through October 12, 2018.

Full study participation required submission of deidentified clinical details for inpatients treated with any antibiotic on a single assessment day, which was specified to be a Tuesday, Wednesday, or Thursday within the study period (October 16, 2018 through January 13, 2019). If sites could not submit data for all inpatients on antibiotics on that single day, a random number generator was used to select a random subset of the hospital's inpatients for entry into the dataset. All patient data were entered by employees of the participating hospital who had access to patient electronic health record (EHR) and antibiotic utilization data as part of their professional duties (e.g., clinical pharmacist). A data entry guide was distributed to all participating sites to ensure consistent data entry.

Patient clinical details included age, sex, race, and inpatient location (e.g., medical floor, intensive care unit). Renal disease was defined as an elevated creatinine on admission or renal failure of any type on the patient's diagnoses, problem list, or admission note. A patient was considered diabetic if diabetes was listed in diagnoses, problem list, or admission note; if the patient was on diabetes medications at the time of their admission; or if the patient had a documented HbA1C ≥6.5%. Colonization or infection with methicillin-resistant *Staphylococcus aureus* and vancomycin-resistant *Enterococcus* was recorded using microbiology, infection control "flags," and problem list data. Penicillin and cephalosporin allergy histories and their associated reactions were identified from the allergy module of the EHR. Specific infection(s) treated, identified by manual chart review, included 19 coded infections (e.g., bacteremia, cellulitis, endocarditis, pneumonia, sepsis) as well as "unknown" and "other." Multiple active infections per patient were permitted. Antibiotics received or pending administration on the assessment day were recorded. Hospital day number was recorded considering the integer difference between the patient's admission date and the assessment day.

This study considered adult inpatients who had cellulitis or skin of soft tissue infection only (without other infections) from acute care hospitals.   The exposure was any race, comparing white and black race. The outcome was antibiotic use.

Statistical Analysis
Numbers and frequencies are reported for categorical variables. Continuous variables are reported as means with standard deviations or medians with interquartile ranges, as appropriate. We examined the relation of race to antibiotic use using generalized estimating equations models to account for clustering by hospital with logit link. In the multivariable models, we considered hospital day, MRSA colonization/infection, and history of penicillin allergy as potentially confounding variables warranting inclusion in the final models *a priori*. All other variables were individually assessed for confounding.

Institutional Review Board
This study was reviewed by the Mass General Brigham Human Research Committee and determined to be exempt/non-human subjects research (Protocol 2018P001722).

© 2021 Wurcel AG et al. *JAMA Network Open.*
